# Prognostic Value of *KRAS* Exon 3 and Exon 4 Mutations in Colorectal Cancer Patients

**DOI:** 10.7150/jca.59193

**Published:** 2021-07-03

**Authors:** Tianan Guo, Yuchen Wu, Dan Huang, Yutong Jin, Weiqi Sheng, Sanjun Cai, Xiaoyan Zhou, Xiaoli Zhu, Fangqi Liu, Ye Xu

**Affiliations:** 1 Department of Colorectal Surgery, Fudan University Shanghai Cancer Center, Shanghai, China.; 2 Department of Oncology, Shanghai Medical College, Fudan University, Shanghai, China.; 3 Department of Pathology, Fudan University Shanghai Cancer Center, Shanghai, China.; 4 Department of Biostatistics and Bioinformatics, Emory University, Atlanta, GA.

**Keywords:** *KRAS exon 3*, * KRAS* exon 4, *KRAS* mutations, colorectal cancer, clinicopathologic features, prognosis

## Abstract

**Background:** The clinical significance of *KRAS* exon 3/4 mutations in colorectal cancer (CRC) remains unclear. We aimed to assess the prognostic value of *KRAS* exons 3 and 4 mutations to determine the necessity for their testing.

**Methods:** *KRAS* mutations in exon 2/3/4 were evaluated in 1816 stage I-IV patients with colorectal adenocarcinoma.

**Results:** The mutation rates of *KRAS* and *KRAS* exons 2, 3, and 4 were 49.0%, 43.0%, 1.9%, and 4.1%, respectively. Univariate survival analysis showed that patients with exon 3 mutation had worse overall survival (OS) compared to those with *KRAS* exon 2 mutation or wild-type *KRAS* (P = 0.044, and P = 0.001). Meanwhile, there was no difference in survival between patients with wild-type *KRAS* and with exon 4 mutation (P = 0.128). In multivariate analysis, *KRAS* mutations in exon 3 and 2 were both independent factors for worse OS (Exon 3, P = 0.032, HR = 1.861, 95% CI: 1.021-3.391; Exon 2, P = 0.049, HR = 1.298, 95% CI: 1.002-1.682). Among the patients with *KRAS* exon 2 mutations, those that had mutations in codon 13 had significantly worse prognosis than those with wild-type *KRAS* (P = 0.001) or *KRAS* codon 12 mutations (P = 0.003).

**Conclusions:** In *KRAS*-mutated CRC, exon 3 mutations predict the worst prognosis, while exon 4 mutations predict the best prognosis. Among *KRAS* exon 2 mutated patients, codon 13 mutations predict worse prognosis than codon 12 mutations. Mutations of different *KRAS* exons should be analyzed separately.

## Introduction

Colorectal cancer (CRC) is the third and second most common malignancy in men and women worldwide, respectively, and the fourth leading cause of cancer-related mortality 1, 2. *KRAS* is one of the first genes to be identified as an oncogene in CRC. Detection of *KRAS* mutations has emerged as an important assessment method for patients with CRC due to its clinical value in predicting prognosis and resistance to targeted therapies 3. *KRAS* mutations are often found in exons 2, 3, and 4, with *KRAS* exon 2 mutations being the most common, accounting for 81-96% of all *KRAS* mutations. The remaining 4-19% of mutations are located in *KRAS* exons 3 and 4 4-6. Despite the lower frequency of mutations in *KRAS* exons 3 and 4, they should not be neglected given the high prevalence of CRC.

Currently, mutations in *KRAS* exon 2 are routinely tested for metastatic CRC in most clinical institutions, and the identification of such mutations has been wildly reported to be associated with poor prognosis and resistance to anti-epidermal growth factor receptor (EGFR) therapy 3, 7, 8. In contrast, mutations in exons 3 and exon 4 of *KRAS* are not widely tested due to their low mutation rate. Thus, the prognostic value of mutations in *KRAS* exon 3/4 remains unclear, and patients harboring *KRAS* mutations in exon 3 or exon 4 are usually combined into one group to meet the number requirements for analysis 5, 9, 10. Further, no consensus has been reached on the clinicopathologic features and prognosis of patients with *KRAS* mutations in exon 3 or exon 4 11-13.

Specific mutations in the *KRAS* gene are closely related to the precise treatment of colorectal cancer. We have previously analyzed the clinicopathologic features and prognostic value of *KRAS*, *NRAS*, and *BRAF* mutations in our cohort 14. The present study aimed to assess the prognostic value of *KRAS* mutations in exons 3 and 4 to elucidate the necessity for their testing as well as to identify the clinicopathologic characteristics of patients harboring these mutations.

## Methods

### Patients

**Figure 1:**
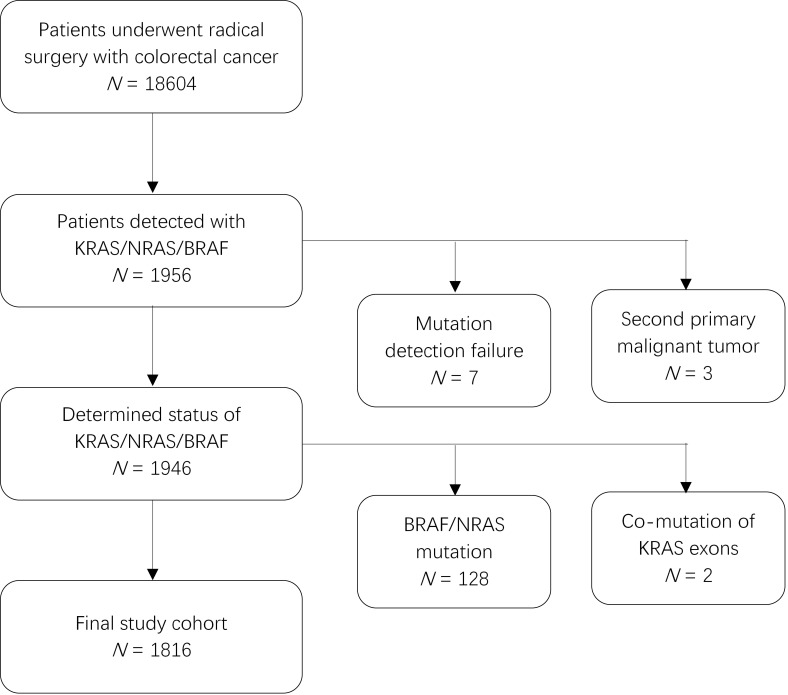
Patient inclusion flowchart.

This was a retrospective study of patients who underwent radical surgery and were pathologically diagnosed with CRC between July 2010 and June 2018. Of the 18604 patients whose tumor tissues were screened for mutations, 1949 patients were identified to have mutations in *KRAS*, *NRAS*, or *BRAF*. We excluded three patients due to a second primary malignant tumor (two with hepatocellular carcinoma and one with lung adenocarcinoma). Further, considering the effect of *BRAF* and *NRAS* mutations on prognosis, we also excluded 128 patients with infrequent mutations in *BRAF* (V600E or non-V600E) or *NRAS* (exon 2/3/4) and 2 patients that harbored *KRAS* mutations in both exons 2 and 3 or in exons 2 and 4. Finally, 1816 patients were included in the analysis (Figure 1). This study was approved by the Research Ethics Committee of the Fudan University Shanghai Cancer Center in China, and all patients provided written informed consent.

### Mutation analysis

Mutation analysis was performed in formaldehyde fixed-paraffin embedded tissues after confirmation by two pathologists in hematoxylin and eosin-stained slides. Genomic DNA was extracted using the QIAamp DNA Mini Kit following the manufacturer's protocol (Qiagen, Valencia, CA, USA). DNA content was quantified using NanoDrop ND-1000 (Nanodrop, Wilmington, DE, USA). Sequencing was performed in 1311 patients. *KRAS* exon 2, 3, and 4; *NRAS* exon 2, 3, and 4; and *BRAF* exon 15 were amplified using the Real-time PCR master mix (TOYOBA, Osaka, Japan) and bidirectionally sequenced via ABI 3730XL and BigDye Terminator v. 3.1 Cycle Sequencing kit (Applied Biosystems, Carlsbad, CA, USA). The positive samples were further confirmed by three independent experiments. For the other 505 patients, amplification refractory mutation system (ARMS) analysis was conducted for mutations in *KRAS* exon 2 (codon 12/13), 3 (codon 59/61), and 4 (codon 117/146) using the AmoyDx *KRAS*/*NRAS*/*BRAF* Mutations Detection Kit (Amoy Diagnostics, Xiamen, China). All experiments were conducted as per the manufacturer's recommendations.

### Statistical Analysis

Statistical analysis was performed using SPSS software version 25.0 (IBM Corporation, Armonk, NY, USA). A two-sided P-value < 0.05 was considered statistically significant. Chi-square tests and Fisher's exact tests were used to compare the categorical variables. For continuous variables, the Kolmogorov-Smirnov test was performed to verify the normal distribution assumptions. The exploratory comparison of normally distributed and non-normally distributed independent groups was performed using t-tests and Mann-Whitney U tests, respectively, for comparison between two groups and using analysis of variance (ANOVA) for comparison between more than two groups. Logistic regression was used for multivariate analysis. Overall survival (OS) was defined as the time between the first surgery and death from any cause. For survival analysis, curves were plotted using the Kaplan-Meier method and analyzed using the log-rank test. Univariate and multivariate analyses to identify prognostic biomarkers were performed using Cox proportional hazard models.

## Results

### Mutational landscape and clinicopathologic characteristics of the patients

The total *KRAS* mutation rate was 49.0% (889/1816), while the *KRAS* exon 2, 3, and 4 mutations rates were 43.0% (780/1816), 1.9% (34/1816), and 4.1% (75/1816), respectively. The mutation analysis results are shown in Table 1. The patients' clinicopathologic characteristics and the results of univariate analysis are shown in Table 2. Meanwhile, the results of multivariate analysis are shown in Table 3. In both univariate and multivariate analyses, a high rate of *KRAS* exon 2 mutation was associated with the following factors: female sex, advanced age, right-colon tumor, high carcinoembryonic antigen (CEA) levels, stage IV tumor, tumor type histology, and extranodal tumor deposit. The *KRAS* exon 3 mutation rate was higher in female patients and in patients with extranodal tumor deposit. Factors associated with a high *KRAS* exon 4 mutation rate included advanced age, tumor type histology, and mucinous carcinoma.

### Survival analysis

**Figure 2:**
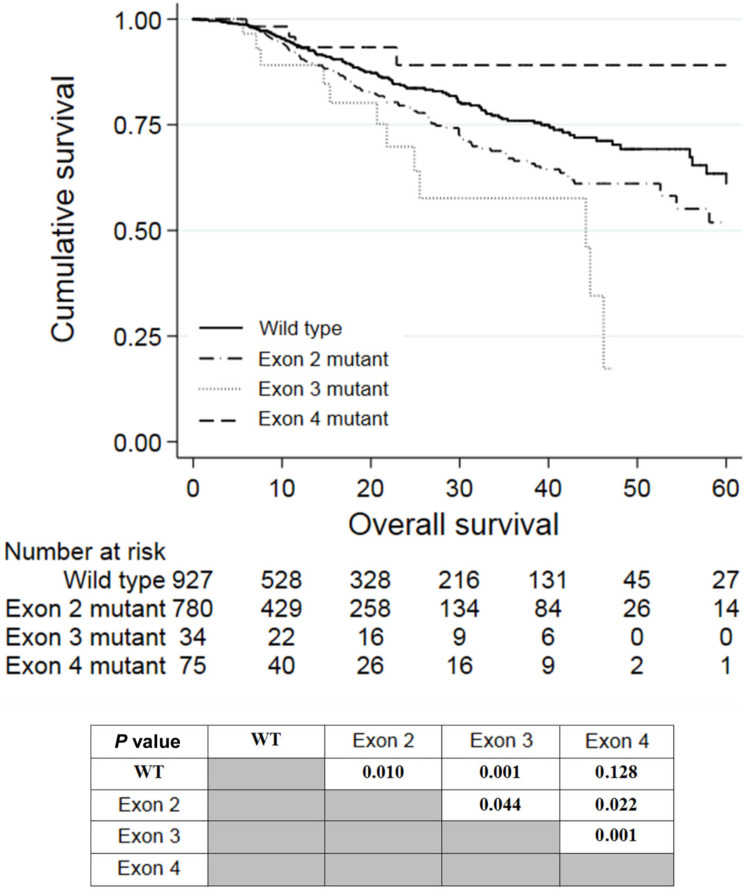
Kaplan-Meier analysis of patients with *KRAS* exon 2, 3, or 4 mutations.

Univariate analysis revealed that a right-colon tumor, larger tumor size, higher CEA levels, neoadjuvant treatment, adjuvant treatment, stage III and stage IV tumor, palliative resection, mucinous carcinoma, poor differentiation, perineural invasion, extranodal tumor deposit, and *KRAS* mutations in exon 2 or 3 were associated with shorter OS. Among these factors, larger tumor size (P = 0.001, HR = 1.073, 95% CI: 1.028-1.120), stage IV tumor (P = 0.027, HR = 3.203, 95% CI: 1.140-9.005), palliative resection (P = 0.001, HR = 2.371, 95% CI: 1.678-3.350), poor differentiation (P = 0.002, HR = 1.520, 95% CI: 1.167-1.978), perineural invasion (P = 0.016, HR = 1.385, 95% CI: 1.062-1.805), extranodal tumor deposit (P = 0.001, HR = 1.573, 95% CI: 1.198-2.066), and *KRAS* mutations in exon 2 (P = 0.049, HR = 1.298, 95% CI: 1.002-1.682) or 3 (P = 0.032, HR = 1.861, 95% CI: 1.021-3.391) were independently associated with worse OS in multivariate analyses. The survival curves of patients with wild-type *KRAS* and *KRAS* mutations in different exons are shown in Figure 2. Patients who harbored *KRAS* exon 2 mutations had poorer prognosis than those with wild-type *KRAS* (P = 0.010) and *KRAS* exon 4 mutations (P = 0.022). Meanwhile, patients with *KRAS* exon 3 mutations had an even worse prognosis than those with exon 2 mutations (P = 0.044). However, patients with *KRAS* exon 4 mutations survived slightly longer than wild-type patients, but the difference was not significant (P = 0.128).

**Figure 3:**
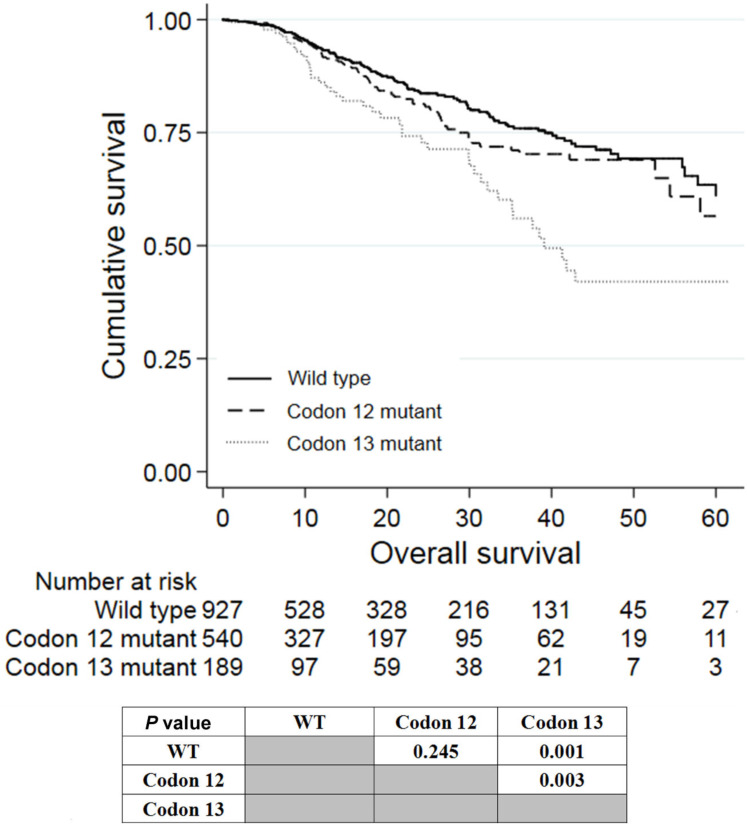
Kaplan-Meier analysis of patients with *KRAS* mutations in codon 12 or 13 of exon 2.

Of the 780 patients with *KRAS* exon 2 mutations, 540 and 189 patients had mutations in codon 12 and 13, respectively. Patients with mutations in codon 13 had worse prognosis than wild-type patients (P = 0.001; Figure 3) and patients with mutations in codon 12 (P = 0.003). Patients with *KRAS* codon 12 mutations tended to have worse prognosis than wild-type patients, although it was not statistically significant (P = 0.245). The survival of patients having codon 59/61 and codon 117/131/146 mutations was not analyzed because of the small number of patients with these mutations.

## Discussion

In this study, we retrospectively analyzed the prognostic value of mutations in *KRAS* exons 2, 3, and 4. We found that patients with *KRAS* exon 3 mutations had the worst prognosis, while patients with exon 4 mutations had the best prognosis. Further analysis of codon 12 and 13 sequences in *KRAS* exon 2 mutation showed that patients harboring mutations in codon 13 had worse prognosis than those with mutations in codon 12. To our best knowledge, this is the first study to (1) report significant differences in prognosis between *KRAS* exon 3 and exon 4 mutations and (2) report worse prognosis of patients with *KRAS* codon 13 mutations compared to those with mutations in codon 12. Further, our cohort of 1816 patients is the largest cohort size in which *KRAS* exon 3/4 mutations have been tested.

Despite evidence that mutations in RAS genes, particularly *KRAS*, play an essential role in predicting resistance to anti-EGFR therapy and worse prognosis in CRC patients, only a few studies have investigated the clinical value of mutations in exons 3 and 4. Information on the clinical relevance of *KRAS* mutations is largely based on *KRAS* exon 2 testing. Although *KRAS* exon 3/4 mutations are associated with clinicopathologic features or patient survival, the necessity of *KRAS* exon 3/4 testing has not been determined to date. Thus, we conducted this single-center retrospective study to explore the impact of *KRAS* exons 3 and 4 mutations on patient survival.

It has been well demonstrated that patients harboring *KRAS* exon 2 mutations do not benefit from anti-EGFR therapy. Lièvre et al. 3 found that *KRAS* exon 2 mutations were associated with resistance to cetuximab and poor prognosis. Amado et al. 8 also reported that *KRAS* exon 2 mutations influence response to panitumumab. Recent clinical trials also showed that patients with *KRAS* exons 3 and 4 mutations lack response to anti-EGFR therapy. However, patients with *KRAS* mutations in exons 3 or 4 were usually analyzed together as one group due to the small cohort size. In the PRIME study, mutations in the exon 3 of *KRAS* were detected in 24 out of 641 metastatic CRC patients, while 36 patients were detected with mutations in exon 4. These patients, along with 48 patients with *NRAS* mutations, were further analyzed as one group 5. Similar situation was also seen in the CRYSTAL study, where 63 patients harboring mutations in *KRAS* exons 3/4 or *NRAS* exon 2/3/4 were categorized into one group 10. These findings formed the basis for international guidelines to recommend that all patients with metastatic CRC should be tested for *KRAS* exon 2/3/4 mutations and that patients detected with mutations in any of these exons should not be treated with anti-EGFR therapy. However, we believe that randomized, controlled trials with larger patient cohorts are needed to determine whether these findings are also representative for patients harboring mutations in exons 3 or 4.

The *KRAS* exon 2/3/4 mutation rates in the current study cohort were consistent with those in two previous studies conducted in relatively large populations 5, 15. In the study by Vaughn et al. 15, *KRAS* exon 2 mutations were detected in 42.4% (900/2121) of their patients, while mutations in exon 3 and 4 were detected in only 3.7% (19/513) and 3.3% (17/513) of 513 exon 2 wild-type patients, respectively. However, in their study, no mutations in codons 59 or 117 were detected, which might explain why their results differ from ours. Similarly, Douillard et al. 5 reported mutation rates of *KRAS* mutations in exons 2, 3, and 4 of 40.1% (440/1096), 3.7% (24/641), and 5.6% (36/641), respectively. *KRAS* codon 61 mutations were also detected in that study, but mutations in codon 59 were not. In an Eastern population, Guo et al. 16 evaluated 353 stage I-IV Chinese CRC patients, and the *KRAS* mutation rates in exons 2, 3, and 4 were 42.2% (149/353), 2.3% (8/353), and 8.2% (29/353), respectively. The differences in mutation rates might be due to the differences in cohort size.

With respect to clinicopathologic features, our study showed that *KRAS* mutations in exon 2/3 were associated with a high rate of positive extranodal tumor deposit, and extranodal tumor deposit was known as a predictor of poor prognosis 17, 18. This might be related to the poor prognosis of patients harboring *KRAS* exon 2/3 mutations. No relevant findings from other studies have been reported yet.

The prognostic value of *KRAS* exon 3/4 remained unclear before the current study 19-21. Similar survival trends of patients with mutations in *KRAS* exon 2/3/4 were reported by Frankel et al. 22 in a cohort of 165 stage IV CRC patients, although the small cohort size did not allow for robust statistical analyses. In most studies, patients with mutations in *KRAS* exon 3/4 were combined for analysis 11-13, which was not reasonable according to our results because mutations in these exons resulted in very different prognosis.

The poor prognosis of patients with *KRAS* exon 2 mutations has been widely reported. However, the prognostic value of mutations in codons 12/13 is controversial. In a study of 1075 stage I-IV CRC patients by Imamura et al. 23, survival analysis showed that mutations in codon 12, but not codon 13, was associated with a worse prognosis than wild-type *KRAS*. Similar results were found by Margonis et al. 24 in a study of 512 stage IV CRC patients. Passot et al. 25 evaluated 524 stage IV CRC patients and reported that patients with mutations in codon 12 or 13 had worse prognosis than *KRAS* wild-type patients. However, there was no significant difference in the prognosis of patients with codon 12 and codon 13 mutations, consistent with the findings of some other studies 13, 26. The varying results in these studies could be caused by the differences in cohort size, data analysis methods, or race.

This study was subject to limitations because of its single-center and retrospective design. Further, the influence of specific amino acid alterations on prognosis was unclear due to the small sample size of the subgroups. Further studies with a larger sample size and longer follow-up are needed to validate our findings. We will focus on response analysis of chemotherapies (irinotecan based or oxaliplatin based) and targeted therapies (cetuximab or bevacizumab) when a sufficient number of cases are accumulated.

In summary, this large-scale retrospective study demonstrated that *KRAS* mutations in exon 3 predict the worst prognosis, while those in exon 4 predict the best prognosis. *KRAS* mutations in codon 13 predict worse prognosis compared to mutations in codon 12. Thus, further studies on treatment efficacy should evaluate patients with *KRAS* exon 3 mutations separately from those with *KRAS* exon 4 mutations.

## Figures and Tables

**Table 1:** Mutations detected in the 1816 *BRAF*/*NRAS* wild-type patients

Mutations	Codons	Amino acid alteration	Number of patients
Wild type			927
*KRAS* exon 2 mutation	Codon 12	p.G12D	174
		p.G12V	121
		p.G12S	36
		p.G12C	26
		p.G12A	15
		p.G12R	8
		Other amino acid alteration	7
		Unknown amino acid alteration	153
	Codon 13	P.G13D	184
		p.G13R	3
		p.G13C	1
		p.G13V	1
	Unknown codon		51
*KRAS* exon 3 mutation	Codon 61	p.Q61H	8
		p.Q61L	7
		p.Q61R	5
		p.Q61K	2
	Codon 59	p.A59T	2
	Unknown codon		10
*KRAS* exon 4 mutation	Codon 146	p.A146T	44
		p.A146V	4
		p.A146N	1
	Codon 117	p.K117D	5
		p.K117N	3
		p.K117R	1
	Codon 131	p.Q131fs	1
	Unknown codon		16

**Table 2:** Univariate analysis of the clinicopathologic features

Variables	Wild type *N* = 927 (%)	*KRAS*		*KRAS* exon 2		*KRAS* exon 3		*KRAS* exon 4	
		Mutant *N* = 889 (%)	*P-*value	Mutant *N* = 780 (%)	*P-*value	Mutant *N* = 34 (%)	*P-*value	Mutant*N* = 75 (%)	*P-*value
**Sex**			**0.001**		**0.003**		**0.020**		0.379
Male	591 (63.8)	501 (56.4)		442 (56.7)		15 (44.1)		44 (58.7)	
Female	336 (36.2)	388 (43.6)		338 (43.3)		19 (55.9)		31 (41.3)	
Age	60.0 (22-89)	62.0 (20-91)	**0.003**	62.0 (20-91)	**0.011**	60.0 (37-90)	0.749	64.0 (34-89)	**0.017**
**Tumor site**			**0.001**		**0.001**		0.140		**0.004**
Rectum	430 (46.4)	429 (48.3)		373 (47.8)		12 (35.3)		44 (58.7)	
Left	321 (34.6)	186 (20.9)		164 (21.0)		11 (32.4)		11 (14.7)	
Right	176 (19.0)	274 (30.8)		243 (31.2)		11 (32.4)		20 (26.6)	
Tumor size	4.0 (0.5-17.0)	4.2 (0.5-22.0)	**0.040**	4.1 (0.5-22.0)	0.089	3.9 (1.5-7.5)	0.933	4.5 (1.2-11.0)	**0.014**
**CEA level**			**0.001**		**0.001**		0.113		0.175
≤5 μg/L	520 (56.1)	411 (46.2)		361 (46.3)		14 (41.2)		(48.0)	
>5 μg/L	407 (43.9)	478 (53.8)		419 (53.7)		20 (58.8)		39 (52.0)	
**T stage**			0.676		0.514		0.148		0.665
T1	31 (3.3)	25 (2.8)		21 (2.7)				4 (5.3)	
T2	126 (13.6)	107 (12.0)		92 (11.8)		3 (8.8)		12 (16.0)	
T3	512 (55.2)	506 (56.9)		453 (58.1)		16 (47.1)		37 (49.4)	
T4	258 (27.9)	251 (28.2)		214 (27.4)		15 (44.1)		22 (29.3)	
**N stage**			0.124		0.111		0.114		0.542
N0	399 (43.0)	351 (39.5)		306 (39.2)		10 (29.4)		35 (46.7)	
N1-2	528 (57.0)	538 (60.5)		474 (60.8)		24 (70.6)		40 (53.3)	
**TNM stage**			**0.002**		**0.001**		0.057		0.588
I	102 (11.0)	74 (8.3)		60 (7.7)		2 (5.9)		12 (16.0)	
II	235 (25.4)	206 (23.2)		180 (23.1)		7 (20.6)		19 (25.4)	
III	310 (33.4)	280 (31.5)		251 (32.2)		9 (26.5)		20 (26.6)	
IV	280 (30.2)	329 (37.0)		289 (37.0)		16 (47.0)		24 (32.0)	
**Histological**			**0.001**		**0.001**		0.596		**0.003**
Ulcer type	662 (71.4)	565 (63.6)		495 (63.5)		27 (79.4)		43 (57.3)	
Tumor type	225 (24.3)	294 (33.1)		257 (32.9)		6 (17.6)		31 (41.3)	
Invasive type	40 (4.3)	30 (3.3)		28 (3.6)		1 (3.0)		1 (1.4)	
**Pathology**			**0.002**		**0.010**		0.460		**0.001**
Adenocarcinoma	851 (91.8)	776 (87.3)		687 (88.1)		30 (88.2)		59 (78.7)	
Mucinous	76 (8.2)	113 (12.7)		93 (11.9)		4 (11.8)		16 (21.3)	
**Differentiation**			**0.023**		0.092		0.157		**0.011**
G3-G4	252 (27.2)	285 (32.1)		241 (30.9)		13 (38.2)		31 (41.3)	
G1-G2	675 (72.8)	604 (67.9)		539 (69.1)		21 (61.8)		44 (58.7)	
**Lymphovascular invasion**			0.811		0.990		0.447		0.095
Negative	604 (65.2)	584 (65.7)		508 (65.1)		20 (58.8)		56 (74.7)	
Positive	323 (34.8)	305 (34.3)		272 (34.9)		14 (41.2)		19 (25.3)	
**Perineural invasion**			0.164		0.097		0.358		0.237
Negative	618 (66.7)	565 (63.6)		490 (62.8)		20 (58.8)		55 (73.3)	
Positive	309 (33.3)	324 (36.4)		290 (37.2)		14 (41.2)		20 (26.6)	
**Extranodal tumor deposit**			**0.011**		**0.013**		**0.024**		0.878
Negative	755 (81.4)	681 (76.6)		597 (76.5)		22 (64.7)		62 (82.7)	
Positive	172 (18.6)	208 (23.4)		183 (23.5)		12 (35.3)		13 (17.3)	

**Table 3:** Multivariate analysis of the clinicopathologic features

Variables	Multivariate analysis							
	*KRAS*		*KRAS* exon 2		*KRAS* exon 3		*KRAS* exon 4	
	*P*-value	OR (95% CI)	*P*-value	OR (95% CI)	*P*-value	OR (95% CI)	*P*-value	OR (95% CI)
Sex (Male)	**0.002**	0.728 (0.598-0.887)	**0.006**	0.752 (0.614-0.922)	**0.035**	0.473 (0.236-0.947)		
Age	**0.001**	1.016 (1.008-1.025)	**0.002**	1.014 (1.005-1.022)			0.002	1.035 (1.013-1.058)
**Tumor site**								
Rectum	Ref	1	Ref	1			Ref	1
Left	**0.001**	0.535 (0.424-0.675)	**0.001**	0.545 (0.428-0.694)			**0.001**	0.328 (0.165-0.651)
Right	**0.008**	1.388 (1.088-1.773)	**0.002**	1.475 (1.151-1.892)			0.750	0.909 (0.505-1.637)
CEA level (>5 μg/L)	**0.001**	1.390 (1.140-1.695)	**0.002**	1.376 (1.121-1.689)				
**TNM stage**								
I	Ref	1	Ref	1				
II	0.160	1.310 (0.899-1.908)	0.068	1.448 (0.973-2.155)				
III	0.244	1.248 (0.860-1.810)	0.066	1.443 (0.975-2.134)				
IV	**0.008**	1.678 (1.143-2.464)	**0.002**	1.868 (1.246-2.801)				
**Histological**								
Ulcer type	Ref	1	Ref	1			Ref	1
Tumor type	**0.001**	1.632 (1.307-2.037)	**0.001**	1.699 (1.352-2.136)			**0.006**	2.009 (1.219-3.311)
Invasive type	0.270	0.751 (0.452-1.248)	0.407	0.803 (0.479-1.348)			0.263	0.314 (0.041-2.385)
Pathology (Mucinous)	**0.038**	1.310 (1.015-1.692)					**0.001**	3.800 (1.974-7.313)
Extranodal tumor deposit	**0.038**	1.310 (1.015-1.692)	**0.034**	1.329 (1.022-1.729)	**0.029**	2.249 (1.087-4.653)		

**Table 4:** Univariate and multivariate analyses of the prognostic variables for OS

Prognostic variables	Univariate analysis		Multivariate analysis	
	*P*-value	HR (95% CI)	*P*-value	HR (95% CI)
Sex (Male)	0.097	0.810 (0.632-1.039)		
Age	0.150	0.993 (0.982-1.003)		
**Tumor site**				
Rectum	Ref			
Left	0.233	1.199 (0.890-1.617)		
Right	**0.001**	1.629 (1.206-2.200)		
Tumor size	**0.001**	1.133 (1.083-1.186)	**0.001**	1.073 (1.028-1.120)
Elevated CEA level	**0.001**	1.862 (1.439-2.408)		
Neoadjuvant treatment	**0.001**	2.062 (1.575-2.700)		
Adjuvant treatment	**0.001**	1.972 (1.401-2.775)		
**TNM stage**				
I	Ref		Ref	
II	0.262	1.881 (0.624-5.668)	0.541	1.412 (0.467-4.270)
III	**0.012**	3.705 (1.330-10.322)	0.114	2.300 (0.818-6.469)
IV	**0.001**	10.058(3.735-27.084)	**0.027**	3.203(1.140-9.005)
Palliative resection^a^	**0.001**	4.310 (3.347-5.552)	**0.001**	2.371(1.678-3.350)
**Histology**				
Ulcer type	Ref			
Tumor type	0.260	0.849 (0.639-1.129)		
Invasive type	0.325	1.285 (0.780-2.116)		
Pathology (mucinous)	**0.001**	1.741 (1.261-2.404)		
Differentiation (G3-G4)	**0.001**	2.271 (1.773-2.920)	**0.002**	1.520 (1.167-1.978)
Lymphovascular invasion	**0.001**	2.100 (1.639-2.691)		
Perineural Invasion	**0.001**	2.042 (1.591-2.622)	**0.016**	1.385 (1.062-1.805)
Extranodal tumor deposit	**0.001**	2.718 (2.116-3.493)	**0.001**	1.573 (1.198-2.066)
*KRAS* exon 2 mutant	**0.010**	1.399 (1.084-1.806)	**0.049**	1.298 (1.002-1.682)
*KRAS* exon 3 mutant	**0.001**	2.518 (1.389-4.567)	**0.032**	1.861 (1.021-3.391)
*KRAS* exon 4 mutant	0.128	0.569 (0.205-1.580)	0.141	0.472 (0.174-1.282)

^a)^ Cases were considered as palliative excision when primary and metastatic lesions were not both radically resected.

## Abbreviations

CRC: colorectal cancer
OS: overall survival
EGFR: epidermal growth factor receptor
ARMS: the amplification refractory mutation system
CEA: carcinoembryonic antigen
OR: odds ratio
CI: confidence interval
HR: hazard ratio
